# Corrigendum: Decreased PPP1R3G in pre-eclampsia impairs human trophoblast invasion and migration via Akt/MMP-9 signaling pathway

**DOI:** 10.3389/ebm.2024.10419

**Published:** 2024-12-13

**Authors:** Huimin Shi, Renyu Kong, Xu Miao, Lingshan Gou, Xin Yin, Yuning Ding, Xiliang Cao, Qingyong Meng, Maosheng Gu, Feng Suo

**Affiliations:** ^1^ Department of Obstetrics, Xuzhou Cancer Hospital, Xuzhou, Jiangsu, China; ^2^ Department of Cell Biology and Neurobiology, Xuzhou Key Laboratory of Neurobiology, Xuzhou Medical University, Xuzhou, China; ^3^ Center for Genetic Medicine, Maternity and Child Health Care Hospital Affiliated to Xuzhou Medical University, Xuzhou, Jiangsu, China; ^4^ Department of Urology, Xuzhou No. 1 People’s Hospital, The Affiliated Xuzhou Municipal Hospital of Xuzhou Medical University, Xuzhou, Jiangsu, China; ^5^ Department of Obstetrics, Xuzhou Maternal and Child Health Hospital Affiliated to Xuzhou Medical University, Xuzhou, Jiangsu, China

**Keywords:** MMP-9, PE, PPP1R3G, trophoblast, invasion

In the original article, there was an error in Figure 3, where the image for the sh-NC group at 12 h was mistakenly duplicated with the image for the sh-PPP1R3G group at 24 h. The corrected Figure 3 is provided below.

**FIGURE 3:**
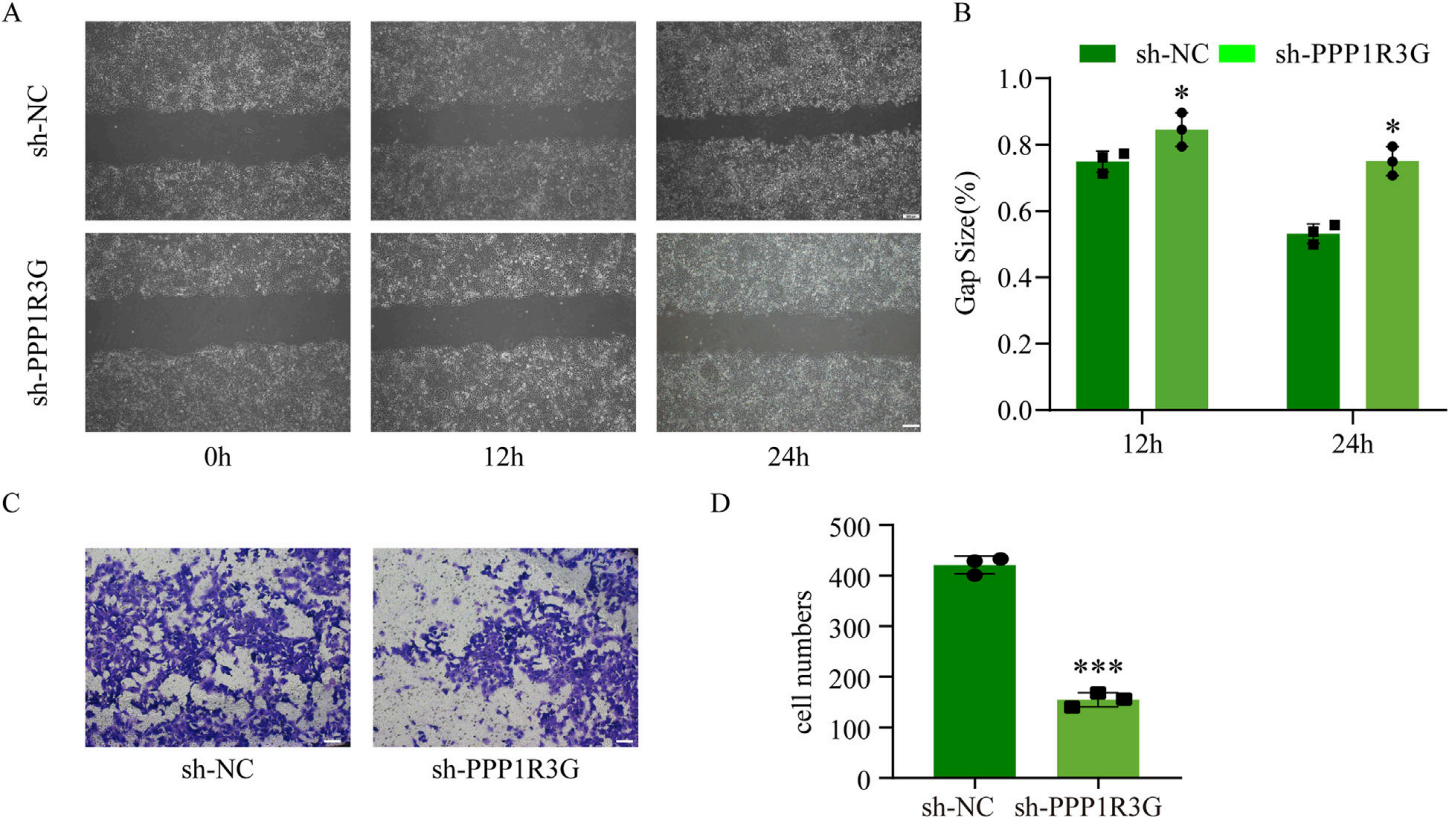
PPP1R3G knockdown significantly inhibits trophoblast invasion and migration. **(A, B)** Representative images of wound-healing assay and quantitative analysis of HTR-8/SVneo cells transfected with sh-PPP1R3G or sh-NC at 0, 12, and 24 h **(C, D)** Representative images of transwell assay and quantitative analysis of HTR-8/SVneo cells transfected with sh-PPP1R3G or sh-NC. Bar = 50 μm. Data are expressed as means ± SEM (n = 3), Student’s t-test, *P < 0.05, ***P < 0.001 versus sh-NC group.

The authors apologize for this error and state that this does not change the scientific conclusions of the article in any way.

